# A HISTORICAL-CONTEXTUAL PERSPECTIVE ON GRANDPARENTS’ ACTIVITIES WITH GRANDCHILDREN

**DOI:** 10.1093/geroni/igad104.0854

**Published:** 2023-12-21

**Authors:** Jill Juris, Amy Rauer

## Abstract

Grandparenting is one of the most positive roles for many older adults, yet there is limited understanding of what activities grandparents and grandchildren actually engage in together or how these may vary based on both context (e.g., residential grandparenthood, grandparents’ marriage) and historical events (e.g., COVID-19). Drawing from multiple theoretical frameworks and methodological approaches, this symposium begins with Flood providing a broad view of time grandparents and grandchildren spend together using large-scale population-level data from IPUMS. Illustrating how such activities may vary based on context, Stephan and Chan provide insights into how activity engagement varies by caregiver status and whether activity engagement with grandchildren is related to grandparents’ perceived roles. Extending this examination of context to consider specific processes, Juris and Zvonkovic will then describe the process of family leisure among grandparent couples by examining how both individuals in a couple experience family leisure with their grandchildren. Providing a historical perspective on these experiences, Fruhauf and colleagues reveal the challenges of raising grandchildren in the context of COVID-19 and the importance of engaging in different and new activities with their grandchildren during this time. Finally, as discussant, Rauer will draw upon Bronfenbrenner’s socioecological framework to illustrate how grandparenting is situated within a complex network of interdependent systems and dynamic contexts. Together, this symposium will highlight the implications of grandparenting for catalyzing development and enhancing well-being across multiple generations.

